# Psychometric validation of the Hospital Anxiety and Depression Scale (HADS) in community-dwelling older adults

**DOI:** 10.1186/s12888-023-05407-2

**Published:** 2023-12-05

**Authors:** Heidi Emly Sivertsen, Anne-Sofie Helvik, Linda Gjøra, Gørill Haugan

**Affiliations:** 1 Department of Public Health and Nursing, Faculty of Medicine and Health Sciences, Norwegian University of Science and Technology (NTNU), Trondheim, Norway; 2 The Norwegian National Centre for Ageing and Health, Vestfold Hospital Trust, Tønsberg, Norway; 3 Department of Psychiatry, Levanger Hospital, Nord-Trøndelag Hospital Trust, Levanger, Norway; 4 Faculty of Nursing and Health Sciences, Nord University, Levanger, Norway

**Keywords:** Aged, Anxiety, Community-dwelling, Depression, Dimensionality, Factor analysis, Independent living, Psychometrics, Reliability, Validity

## Abstract

**Objectives:**

The Hospital Anxiety and Depression Scale (HADS) is commonly used to measure anxiety and depression, but the number of studies validating psychometric properties in older adults are limited. To our knowledge, no previous studies have utilized confirmative factor analyses in community-dwelling older adults, regardless of health conditions. Thus, this study aimed to examine the psychometric properties of HADS in older adults 70 + living at home in a large Norwegian city.

**Methods:**

In total, 1190 inhabitants ≥ 70 (range 70 – 96) years completed the HADS inventory in the population-based Trøndelag Health Study (HUNT), termed “HUNT4 70 + ” in Trondheim, Norway. Confirmatory factor analyses were performed to test the dimensionality, reliability, and construct validity.

**Results:**

The original two-factor-solution (Model-1) revealed only partly a good fit to the present data; however, including a cross-loading for item 6_D_ (*“I feel cheerful”*) along with a correlated error term between item 2_D_ (*“I still enjoy the things I used to enjoy”*) and 12_D_ (*“I look forward with enjoyment to things*”) improved the fit substantially. Good to acceptable measurement reliability was demonstrated, and the construct validity was acceptable.

**Conclusions:**

The HADS involves some items that are not reliable and valid indicators for the depression construct in this population, especially item 6 is problematic. To improve the reliability and validity of the Norwegian version of HADS, we recommend that essential aspects of depression in older adults should be included.

## Introduction

Depression among older people, either reported as major depressive disorder (MDD) or clinically significant depressive symptoms (CSDS), is associated with decreased quality of life (QOL) [1], increased comorbidity with physical illness, reduced emotional, cognitive, and physical functioning in daily activities, increased risk of dementia [2] and increased need for help, and risk of death [3–5]. Accordingly, early identification and treatment of depressive symptoms is important in several ways; not only for the individual having the symptoms, but also for the family, and the associated health costs to the society. Demographical changes worldwide involve that the segment of older people is significantly increasing, and most will stay in their homes.

Older adults aged 70 + living at home may have multiple diseases [6] accompanied by impaired physical [7–11] and cognitive functionality [3, 10, 12]. In older people, less characteristic symptoms of depression do often appear; compared to younger age groups, low mood and sadness are less prevalent [9, 10], whereas somatic symptoms, painful conditions, and physical disability, along with anxiety and cognitive impairment are much more prevalent [8]. As a result of this uncharacteristic symptomatology, depression is less frequently diagnosed and treated among older adults [4, 5, 8, 12–14]. In addition, many community-dwelling older adults who meet the diagnostic criteria for depression do not seek health care for their symptoms; less than half have contact with the health service, and barely 10% receive effective treatment [12]. To better identify and meet symptoms of depression in older people at home, access to a valid and reliable tool assessing depressive symptoms among older community-dwelling adults is crucial.

### Background

Depression is prevalent among older adults (≥ 70 years) worldwide, with a point estimate of depressive disorders of 5.4%, including both MDD and CSDS [15]. Furthermore, a recent review based on 20 studies, among which 14 were from community-dwelling settings, found a pooled point prevalence of 13.3% for MDD among older adults [16]. Two recent systematic reviews primarily based on studies of community-dwelling older adults found a pooled point prevalence of 28.4% and 31.7% for CSDS, with a large variation between countries [17, 18]. These two reviews explained the large variety of CSDS estimates by cultural differences along with differences in sample characteristics, methodology, and screening tools used in the included original studies [17, 18].

Compared to younger age groups, older adults report a higher frequency of anxiety [12, 19]. In 2019, the estimated prevalence of anxiety disorders (a combined estimate of all subtypes) among adults aged ≥ 70 years was 4.4% [15]. A recent review among community-dwelling adults ≥ 55 years old revealed a pooled prevalence of anxiety disorders and anxiety symptoms of 5.4% and 7.9%, respectively [20]. Generalized anxiety disorder (GAD) is the most prevalent, leading to a high risk of death, even higher when accompanied by depression [21]. Similar to depression, anxiety symptoms are less characteristic among older adults than in younger age groups [20]; for instance are worries about health, sleep disturbances, and reduced reassurance-seeking behaviors more common symptoms of anxiety in older populations [19].

Moreover, compared to younger adults, older people are less likely to report and seek help for their symptoms, have less knowledge regarding anxiety disorders and available treatment, and face barriers to treatment such as stigma, cost, transportation, and mobility [22]. Ageism, making older people regularly experience prejudice and discrimination in health care, is another reason for not seeking treatment. Hence, ageism might contribute to heightened levels of anxiety and depression [23]. Furthermore, multimorbidity may lead to overlooking clinically relevant symptoms of depression and anxiety. To uncover symptoms of depression and anxiety among older community-dwelling people, easy access to and use of reliable and valid screening measure are needed.

Possibly, the lack of treatment for clinically relevant symptoms of depression and anxiety represents an alarming concern to the healthcare system and society; health politics highlights the need for health promotion interventions to keep older people in their own homes as long as possible. Undetected issues of depression and anxiety may cause reduced mental and physical functionality and increased risk of dementia and other diseases [1, 3–5], all of which trigger a need for professional health care.

In many older people, depressive symptoms can be difficult to distinguish from age-related symptoms such as sleep disturbance, psychomotor changes, concentration disturbance, changes in nutrition and digestive functioning, and fatigue [4]. Furthermore, anxiety and depression may co-exist [23]. Hence, differentiating between anxiety and depressive symptoms is complicated, caused by overlapping and coinciding symptoms. For example, fatigue, physical symptoms, and negative ruminations have the same clinical features in both diseases [23].

The Hospital Anxiety and Depression Scale (HADS) is commonly used in epidemiological research to estimate the prevalence of clinically significant anxiety symptoms and CSDS among adults [17] and in clinical settings to detect clinically relevant symptoms [1, 24, 25]. HADS was developed to detect clinically relevant symptoms of both anxiety and depression without including any physical symptoms. Thus, the HADS was developed to distinguish between depression and anxiety symptoms. However, since anxiety and depression symptoms may co-exist, we expect these two constructs to correlate to some extent.

The HADS is widely used and tested with satisfactory psychometric properties [26, 27]. However, only a handful of studies have evaluated HADS using confirmative factor analysis (CFA) in community-dwelling, non-clinical samples of older adults [26, 28, 29]; these studies support the original two-factor structure. Problems with items loading on both anxiety and depressive symptoms (cross-loadings) are reported, though [26]. Furthermore, a psychometric evaluation of HADS in a clinical sample of veterans utilizing CFA reported a three-factor structure showing the best fit [30]; the third factor was entitled “dealing with the inability to feel pleasure.” Thus, the dimensionality of HADS is questioned [31].

Initially, the HADS was developed for adults receiving treatment for physical health problems in general hospitals [31]. Still, HADS is used among community-dwelling and older people; evidence suggests a lower cut-off for older adults than among younger populations [32]. This may indicate that HADS, developed for younger hospitalized age groups, might have psychometrical traits, making it less suitable for older adults [20].

Utilizing principal component analysis (PCA), three studies have assessed the dimensionality of the Norwegian version of HADS: one assessed older adults admitted to somatic hospitals [33]. In comparison, the two others did not explicitly study older adults [34, 35]. Nevertheless, using only PCA for psychometrical evaluation implicates several limitations. According to the literature, the CFA approach is needed to achieve a robust test of a scale's dimensionality, composite reliability, and construct validity [36]. One study tested the psychometrics of HADS among older adults in nursing homes [37], reporting that several items revealed low reliability and validity in this population. The authors related these findings to characteristics of the nursing home population, such as several chronic diseases, symptom severity, losses of functionality, loneliness, and dependency of care [38]. The characteristics of community-dwelling older people ≥ 70 years living at home differ compared to the nursing home population. Therefore, a psychometric study of HADS among older adults aged 70 + living at home is required. Concerning early detection and treatment of anxiety and depressive symptoms among community-dwelling older people, a valid and reliable scale is highly needed.

### Aims

To date, the dimensionality, internal consistency, construct validity, and homogeneity of the Norwegian version of the HADS have not been assessed using CFA among community-dwelling older adults ≥ 70 years. Therefore, this study aims to examine the psychometric properties of the HADS among older adults ≥ 70 living at home in Norway; dimensionality, reliability, and construct validity are interrelated measurement properties and are thus investigated in this study. The hypotheses are:

- H_1_: The original two-factor model of HADS shows a good fit to the present data,
- H_2_: HADS demonstrates good reliability,
- H_3_: HADS shows good construct validity,
- H_4_: HADS correlates significantly and negatively with QOL and.
- H_5_: The anxiety and depression factors perform as two distinct concepts.

The hypotheses are based on theory and evidence [1, 17, 23–25]; we hypothesized that the original measurement model of HADS shows a good fit to our data [26, 27], good reliability and validity [26, 27, 31], comprises two distinct concepts [23] which correlate positively with each other and negatively with QoL. It is rational that when anxiety and depression increase, QOL decreases. To sum up, H_1,_ H_4_, and H_5_ are chosen according to the theoretical assumption that HADS with anxiety and depression are two distinct concepts, distinctively different from each other and QOL, and therefore, we assume that these concepts provide good reliability and validity (H_2_ and H_3_)_._

## Methods

### Study participants and procedures

During 2017–2019, persons aged ≥ 70 were recruited from one randomly selected district in Trondheim, county of Trøndelag in Central Norway, as a part of the fourth wave of The Trøndelag Health Study (HUNT). HUNT is a population-based cross-sectional study [39]. The HUNT study comprises questionnaires, e.g., HADS, clinical measurements, and collections of biological samples. Older age is defined in HUNT as 70 years and above and is linked to retirement for public sector employees, which is 70 years.

In total, 4667 community-dwelling inhabitants living at home in Trondheim were invited to participate, of whom a total of 1486 (response rate of 31.8%) persons 70 years or older (55.3% women and 44.7% men) participated in the regular protocol and responded to the HADS questionnaire and demographical questions (Q1 and Q2). These participants also underwent a comprehensive clinical evaluation.

The inclusion criteria for the present study were: (1) living at home, (2) aged 70 years or older, (3) without dementia, and (4) having responded to all 14 HADS items since this is considered best practice utilizing CFA. In total, 126 participants were diagnosed with a dementia diagnosis, and 170 participants lacked one or more responses on HADS; all of these were excluded from the analyses, giving an effective sample N = 1190.

### Assessment

Sociodemographic characteristics measured were age, sex (male, female), cohabiting status (“no, I live alone” or “yes, with a spouse/partner”), and educational attainment (primary and lower secondary school 9–10 years, academic or vocational school 1 or 2 years, academic or vocational school 3 years, vocational school/apprentice 3–4 years, college or university < 4 years, college, or university ≥ 4 years).

Medical conditions were reported using self-reported items regarding a history of asthma, diabetes, and heart attack. Functional impairment in daily life > 1 year was self-reported using one item (no or yes). Lastly, care support last year in terms of home care, in-home nursing care, and/or hospitalization in nursing homes were self-reported (yes/no).

Global quality of life (QoL) was assessed with one item: “Thinking about your life at the moment, would you say that you by large are satisfied with life, or are you mostly dissatisfied?” The item was scored on a 7-point scale ranging from very satisfied to very dissatisfied.

The HADS consists of 14 items, including subscales for anxiety (HADS-A; seven items) and depression (HADS-D; seven items). The items are scored on a four-point scale ranging from totally disagree to agree totally. Each item is rated from 0–3, where higher scores indicate more severe anxiety and/or depressive symptoms. The maximum score on each subscale is 21, ranging from 0–7 (normal), 8–10 (mild disorder), 11–14 (moderate disorder), and 15–21 (severe disorder) [31].

To increase acceptability and prevent individuals from feeling tested for mental disorders, symptoms of severe psychopathology have been excluded, which makes HADS more sensitive to milder psychopathology [37, 40]. According to the International Classification of Diseases (ICD-10), five of seven items in HADS-D focus on lack of positive feelings and cover only two of three main criteria for depression; physical symptoms such as loss of energy, sleep- and appetite disturbances are not covered.

### Statistical analysis

The analyses were conducted using the IBM Statistical Package for the Social Sciences Version 28 software [41] and the Stata 17 software package [42]. Confirmatory Factor Analysis (CFA) represents a more accurate evaluation of the psychometric properties of the scales used. In this study, the model fit adequacy was assessed by χ^2^-statistics and conventional fit indices: χ^2^-statistics, the Root Mean Square Error of Approximation (RMSEA), and the Standardized Root Mean Square Residual (SRMR) with values < 0.10 are acceptable, and values ≤ 0.05 indicates a good fit [43, 44]. Further, the Comparative Fit Index (CFI) and the Tucker-Lewis Index (TLI) with acceptable fit at 0.95 and a good fit at 0.97 [43–46]. According to Hair et al. [47], skewness and kurtosis should be below an absolute value of 2.0 (standardized); this was the case for all items (data not shown), indicating that both skewness and kurtosis were significant. Therefore, the Satorra-Bentler corrected χ^2^ which is the correct asymptotic mean even under non-normality, is reported [48].

CFA is well known to be sensitive to sample size [49]; the larger the samples, the bigger the chi-square. A consequence may be that well-fitting models are rejected because the chi-square is too high, resulting from a larger sample size more than the real model fit. The present N is 1190, which is considered large. Therefore, we planned to randomly split the dataset into two equally sized parts to check for model fit in each part, termed Sample 1 and Sample 2; all models involved in this study were tested in both samples. Consequently, the original two-factor solution was tested in the total sample as well as in the two sub-samples.

Since we aimed to find the best fitting model, i.e., a model that represents the observed data of older home-dwelling adults in the best manner, we first tested the original model and then, after, other models based on the findings in this original model. Thus, as stated in the aim section*,* this study investigates the dimensionality, reliability, and construct validity of the HADS among community-dwelling adults 70 years and older; dimensionality, reliability, and construct validity are interrelated measurement properties. *Dimensionality* concerns the homogeneity of a scale’s items [49], indicating if the included items match the defined construct. Depression and anxiety have been seen to correlate strongly but are still considered different constructs. *Reliability* encompasses a scale’s consistency and lack of error [50]. To assess the items' internal consistency, the reliability coefficients of Cronbach’s alpha (α) and composite reliability (ρ_c_) were utilized. Finally, *construct validity* implies various aspects, such as convergent, discriminant, and content validity. In this study, convergent and discriminant validity denote if HADS relates with other constructs as expected, while content validity embraces whether the 14 items adequately represent the theoretical content of the anxiety and depression constructs involved in HADS. Taken together, this is whether the included items cover the theoretical definition they are aimed to represent [51]. If the wording of items is too similar, Cronbach’s alpha, content validity, and dimensionality will be falsely improved. In consequence, the average correlation among items increases, and therefore also coefficient alpha; however, without adding substantially to the content validity of the scale. Obviously, to tap into the same construct, some similarity/correlation is needed. Nevertheless, items simply representing a rephrasing of other items are redundant.

## Results

### Sample characteristics

The sample of 1190 adults were aged between 70–96 years with a mean of 76.5 (SD = 5.3); 644 (54.1%) were women (mean age 76.7) while 546 (45.9%) were men (mean age 76.2) (Table 1). Furthermore, 325 (27.3%) had completed higher education (≥ 4 years of university/college). About 50% had a physical or mental long-term illness, injury, or a disorder that impaired their daily functioning (47.1%), only a few had any in-home care (4.7%), in-home nursing care (4.5%) and/or had been admitted to a nursing home for a period during the last year (3.3%).

**Table 1:** Demographic characteristics of the study population by gender

		**Total (*N* = 1190)**	**Women (*n* = 644, 54%)**	**Men (*n* = 546, 46%)**	***p*-value**
Age, M (SD)		76.5 (5.3)	76.7 (5.4)	76.2 (5.1)	0.004^a^
Cohabiting status, n (%)		709 (59.5)	299 (25.2)	410 (34.5)	< 0.001^b^
Education n (%)					< 0.001^b^
Primary school		115 (9.7)	87 (13.5)	28 (5.1)	
Academic or vocational school 1 or 2 years		190 (16)	138 (21.4)	52 (9.5)	
Academic or vocational school 3 years		125 (10.5)	77 (12)	48 (8.8)	
Vocational school/apprentice 3–4 years		132 (11.1)	56 (8.7)	76 (13.9)	
University/college < 4 years		303 (25.5)	148 (23)	155 (28.4)	
University/college ≥ 4 years		325 (27.3)	138 (21.4)	187 (34.2)	
Morbidity, *n* (%)					
Asthma		125 (10.5)	84 (13)	41 (7.5)	0.001^b^
Diabetes		92 (7.7)	39 (6.1)	53 (9.7)	< 0.005^b^
Heart attack		102 (8.6)	36 (5.6)	66 (12.1)	< 0.001^b^
Impaired function in daily life > 1 year		560 (47.1)	325 (58)	235 (42)	< 0.005
Use of health services, last year, *n* (%)					
Home care		56 (4.7)	37 (5.7)	19 (3.5)	n.s^b^
In-home nursing care		53 (4.5)	35 (5.4)	18 (3.3)	n.s^b^
Hospitalized in a nursing home		39 (3.3)	24 (3.7)	15 (2.7)	n.s^b^
Overall global QOL		1172 (98.5)	636 (98.7)	536 (98.2)	< 0.001^b^

HADS (Hospital Anxiety and Depression Scale)

*n.s* not significant

^a^Independent sample t-test,

^b^Pearson chi-square test

Looking at those who were excluded, these were significantly older (mean age 80.1; SD = 7.1 years), more often female (60%), and with less education (≥ 4 years of university/college; 9.3%).

### HADS item score statistics

The mean anxiety and depression scores were 3.4 (SD = 2.9) and 3.0 (SD = 2.5), respectively (Table 2). The internal consistency of the anxiety and depression constructs (Table 2) was good (α_anxiety_ = 0.79 =) or acceptable (α_depression_ = 0.66). Composite reliability (ρc) displayed values between 0.65–0.78 (Table 3); values ≥ 0.60 are acceptable, whereas values ≥ 0.70 are good [43, 47]. Higher symptom scores on HADS correlated significantly with poorer QOL scores (Table 2), supporting convergent validity (H_3_, H_4_, and H_5_).

**Table 2:** Means, Standard deviation (SD), and Cronbach’s alpha for the Norwegian version of the Hospital Anxiety and Depression Scale (HADS)

	**Response**^a**^								
**Items**	0 (%)	1 (%)	2 (%)	3 (%)	**Total**	**Mean**	**SD**	**Cronbach alpha (α)**	**QOL Pearson r**
1^A^ I feel tense or 'wound up'*	76.6	20.6	2.4	0.5	1190	.27	.52		.372^**^
2^D^ I still enjoy the things I used to enjoy	59.2	37.8	2.4	0.7	1190	.45	.58		.405^**^
3^A^ I get a sort of frightened feeling as if something awful is about to happen*	53.9	31.1	10.9	4.1	1190	.65	.83		.274^**^
4^D^ I can laugh and see the funny side of things	77.7	19.8	2.3	0.3	1190	.25	.50		.286^**^
5^A^ Worrying thoughts go through my mind*	65.4	25.1	7.6	1.9	1190	.46	.72		.321^**^
6^D^ I feel cheerful*	69.6	25.4	4.7	0.3	1190	.36	.59		.392^**^
7^A^ I can sit at ease and feel relaxed	55.6	40.3	4.0	0.1	1190	.49	.58		.285^**^
8^D^ I feel as if I'm slowed down*	32.1	56.1	8.3	3.5	1190	.83	.72		.197^**^
9^A^ I get a sort of frightened feeling like 'butterflies' in the stomach	59.0	39.0	1.8	0.3	1190	.43	.54		.263^**^
10^D^ I have lost interest in my appearance*	72.3	21.4	4.2	2.1	1190	.36	.66		.143^**^
11^A^ I feel restless as if I must be on the move*	37.9	47.7	12.8	1.6	1190	.78	.72		.172^**^
12^D^ I look forward with enjoyment to things	61.6	28.4	9.0	1.0	1190	.49	.70		.356^**^
13^A^ I get sudden feelings of panic*	76.4	20.7	2.2	0.8	1190	.27	.54		.216^**^
14^D^ I can enjoy a good book or radio/TV program	83.2	14.0	1.9	0.9	1190	.21	.51		.087^**^
Total A					1190	3.35	2.9	.79	.408^**^
Total D					1190	2.95	2.5	.66	.450^**^
Total A + D					1190	6.30	4.6	.80	.501^**^

*N* = 1190. *A *= anxiety, and D = depression

^*^Items starred are reverse scored

Due to the elevation rules, the total percentages could be higher than 100%. ^**^p < .01

^a^Items were scored on a four-point scale ranging from totally disagree to agree totally. A-response: 0 = ‘Not at all’, 1 = ‘Not very often’, 2 = ‘Quite often’ or 3 = ‘Very often’ and D-response: 0 = ‘Most of the time’, 1 = ’Sometimes’, 2 = ‘Not often’ or 3 = ‘Not at all’. The standard scoring algorithm was used for A = sum of items 1*, 3*, 5*, 7, 9, 11*, 13*; and for D = sum of items 2, 4, 6*, 8*, 10*, 12, 14

**Table 3:** Goodness-of-fit indices for HADS measurement models: Model-1^a^, Model-2^b^, Model-3^c^

Fit Measure	Model-1	Model-2	Model-3	Model Anxiety	Model Depression
	2-factors	2-factors	2-factors	**1-factor**	**1-factor**
χ^2^ *Satorra Bentler*	395.010	317.951	296.919	61.695	84.264
p-value	< 0.0001	< 0.0001	< 0.0001	< 0.0001	< 0.0001
$\frac{{{\varvec{x}}}^{2}}{{\varvec{d}}{\varvec{f}}}$ *Satorra Bentler*	5.20 Df = 76	4.18 Df = 75	4.01 Df = 74	4.41 Df = 14	4.52 Df = 14
RMSEA	0.059 (CI: 0.054–0.065)	0.052 (CI: 0.046–0.058)	0.050 (CI: 0.044–0.056)	0.054 (CI: 0.040–0.068)	0.065 (CI: 0.052–0.079)
p-value *(close fit test)*	0.004	0.262	0.452	0.312	0.030
SRMR	0.052	0.042	0.040	0.029	0.38
CFI	0.91	0.93	0.94	0.97	0.94
TLI	0.89	0.92	0.92	0.96	0.92
^d^Composite Reliability	ρ_anxiety_ 0.78	0.78	0.78	0.78	-
	ρ_depression_ 0.70	0.68	0.65	-	0.70

*N* = 1190

^a^**Model-1** = original 2-factor-model with 14 items

^b^**Model-2 = **Model-1 including a path from item 6_D_ to ANXIETY

^c^**Model-3** = Model-2 including a correlated error between item 2_D_ and 12_D_*.* *RMSEA* Root Mean Square Error of Approximation, *SRMR* Standardized Root Mean Square Residual, *CFI* Comparative Fit Index, *TLI * Tucker-Lewis Index

^d^Composite Reliability = $${\uprho }_{\mathrm{c}}\frac{{(\sum \lambda )}^{2}}{[ ({\sum \lambda )}^{2}+ \sum \left(\uptheta \right) ]}$$

### Confirmatory Factor Analysis (CFA)

This study aimed to test the psychometrics of the HADS among community-dwelling people ≥ 70 years. Consequently, first, we tested the original two-factor solution (H_1_, H_2_, and H_3_), including 14 items. This solution was termed Model-1; the factor loadings (λ) ranged between 0.32 and 0.72, followed by multiple squared correlations (R2) from 0.10 to 0.52; this range of factor loadings was pretty much the same in all estimated models. Three items belonging to the depression construct; 8_D_ (“I feel as I’m slowed down”), 10_D_ (“I have lost interest in my appearance”) and 14_D_ (“I can enjoy a good book or a TV program”) revealed low loadings of 0.34, 0.32 and 0.37, respectively, explaining 12%, 11% and 13% of the variance of the depression construct. The fit indices indicated misspecification: χ^2^ = 395.010, *p* = 0.00001, df = 76, χ^2^/df = 5.20, RMSEA = 0.059, *p*-close = 0.04, SRMR = 0.052, CFI = 0.91, TLI = 0.89 (Table 3). The RMSEA, which is an estimate of approximate fit was acceptable, while the χ^2^ was much too high. For an acceptable fit, the χ^2^/df should be ≤ 3.0, and ≤ 2 for a good fit. Further, the CFI and TLI were too low, all of which indicated misspecification. Exploring the normalized residuals, 23 residuals were significant, with item 6_D_ (*“I feel cheerful “*) involved in several highly significant estimates. Hence, we scrutinized the modification indices (MI) presenting some extremely high values; item 6_D_ exposed an extremely high MI = 77.29 with the anxiety factor and an MI = 30.374 with item 5_A_ (“*Worrying thoughts go through my mind* “). Also, item 2_D_ (*“I still enjoy the things I used to enjoy”*) and 12_D_ (*“I look forward with enjoyment to things*”) demonstrated an exceptionally high MI = 44.383. In total, 16 MIs were ≥ 10.

To further test the original model, we looked at one of the two factors at a time: anxiety and depression, both including 7 items each, demonstrated a too high χ^2^, while the other indices were good to acceptable (*Anxiety*: χ^2^ = 61.695, *p* = 0.00001, df = 14, χ^2^/df = 4.41, RMSEA = 0.054, *p*-close = 0.320, SRMR = 0.029, CFI = 0.97, TLI = 0.96; *Depression*: χ^2^ = 84.264, *p* = 0.00001, df = 14, χ^2^/df = 6.02, RMSEA = 0.065, *p*-close = 0.30, SRMR = 0.038, CFI = 0.94, TLI = 0.92) (Table 3). Composite reliability was good, showing estimates of ρ_Anxiety_ = 0.78 and ρ_Depression_ = 0.70. Both factors revealed a too-high chi. This often causes high modification indices (MIs), indicating cross-loadings and significant correlations among error terms. Hence, we considered the theoretical content of the items.

It is plausible that if one is feeling cheerful, one is improbable to feel anxiety at the same time, and vice versa. Thus, it is theoretically meaningful that feeling cheerful (item 6_D_) and feeling anxious correlate negatively. Accordingly, in Model-2 we included a path (cross-loading) from item 6_D_ (“*I feel cheerful”)* to the Anxiety construct, which improved the fit considerably: χ^2^ = 317.951, p = 0.0001, df = 75, χ^2^/df = 4.24, RMSEA = 0.046, p-close = 0.262, SRMR = 0.042, CFI = 0.93, TLI = 0.92. However, the fit was still not good.

**Fig. 1:**
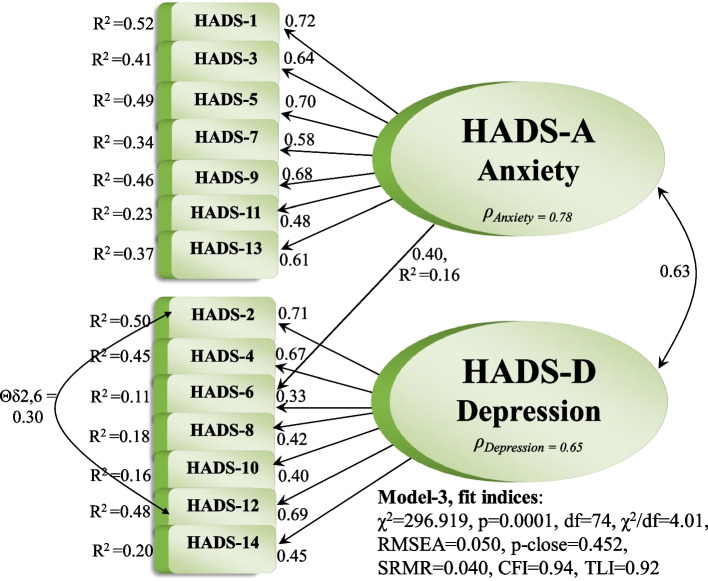
Model 3 – the best fitting measurement model

An extremely high MI between the items 2_D_ (*“I still enjoy the things I used to enjoy”*) and 12_D_ (*“I look forward with enjoyment to things*”) was uncovered. It is rational that still enjoying things and looking forward to things with enjoyment correlate. Therefore, we included a correlated error term between item 2_D_ and 12_D_ which further improved the fit in Model-3: χ^2^ = 296.919, p = 0.0001, df = 74, χ^2^/df = 4.01, RMSEA = 0.050, p-close = 0.452, SRMR = 0.040, CFI = 0.94, TLI = 0.92 (Fig. 1). For Model-3, composite reliability (ρ_c_) was 0.78 for the anxiety subscale and 0.65 for the depression subscale. Still, 14 normalized residuals were significant, asking for several cross-loadings and correlated errors.

Therefore, we checked if a unidimensional solution termed Model-4 would fit better, though revealing an exceedingly bad fit: χ^2^ = 840.194, p = 0.0001, df = 77, χ^2^/df = 10.91, RMSEA = 0.091, p-close = 0.0001, SRMR = 0.070, CFI = 0.78, TLI = 0.74. However, internal consistency was good ρ_HADS_ = 0.81. Hence, the misspecification was possibly caused by error covariances. Consequently, we turned to Model-3 (Fig. 1), again scrutinizing the MI values. The items 8_D_, 12_D_ and 14_D_ displayed MIs > 15. These items also displayed low loadings, indicating poor reliability as indicators for the depression construct. Possibly, removing some of these items would improve the model fit. Nonetheless, composite reliability was ρ_Depression_ = 0.65; hence, removing items would cause a weak construct with even lower reliability.

All tested models revealed a chi-square indicating misspecification. However, as already stated, it is well known that chi-square as a model fit index has limitations. First and foremost, chi-square is sensitive to sample size. The present sample is large (*N* = 1190). Therefore, we randomly split the data into two equally sized parts (*N* = 595) termed Sample 1 and Sample 2, representing a sample size more suitable for SEM (structural equation modeling) [36, 47, 52]. We tested the original HADS (Model-1) in both Sample 1 and Sample 2. Except the chi-square showing better values, the fit indices demonstrated a similar pattern as in the total sample: Sample1 χ^2^ = 225.391, *p* = 0.0001, df = 76, χ^2^/df = 2.97, RMSEA = 0.058, p-close = 0.074, SRMR = 0.057, CFI = 0.90, TLI = 0.88, ρ_Anxiety_ = 0.76 and ρ_Depression_ = 0.67. Sample2 χ^2^ = 257.228, *p* = 0.0001, df = 76, χ^2^/df = 3.38, RMSEA = 0.063, p-close = 0.005, SRMR = 0.053, CFI = 0.91, TLI = 0.90, ρ_Anxiety_ = 0.79 and ρ_Depression_ = 0.73. Loadings ranged between 0.31 and 0.67 for Sample1 and between 0.29 and 0.77 for Sample2.

## Discussion

According to the European Commission’s Green paper on mental health [53], depression is one of the most prevalent mental health problems facing European citizens today. The incidence of depression with increasing age is stated [15]; simultaneously the number of adults over 70 years is globally expected to increase in the coming decades [54]. Hence, access to a valid and reliable scale assessing anxiety and depression among older community-dwelling adults is highly warranted. Therefore, the present study aimed to evaluate the psychometric properties of HADS among community-dwelling older Norwegians ≥ 70 years. In doing this, we tested five hypotheses. The present sample included 1190 older adults, with a mean age of 76.5 years. To the authors’ knowledge, no previous studies have examined the psychometric properties of HADS in a Norwegian population among community-dwelling older adults using CFA.

The CFA approach eliminates the need to summate scales because the SEM programs such as STATA compute latent construct scores for each respondent. This process allows relationships in the model tested to be automatically corrected for error variance, a fundamental strength of CFA in construct validation. Thus, the resulting estimates are adjusted for measurement error [36, 47]. In this study, the original HADS (Model-1) version showed only partly a good fit. In particular, the chi-square demonstrated extremely high values, indicating misspecification. However, utilizing the chi-square as a model fit index relates to some limitations. As already stated, chi-square is sensitive to sample size: a misfit may be trivial, but with larger samples, the p-value decreases, followed by higher estimates [52]. This means that in practice, the chi-square test is “not always the final word in assessing fit” [55]. The present sample size is large (*N* = 1190), revealing extraordinarily high estimates for the chi-square. When splitting the file into two parts, giving a sample size *N* = 595, the chi-square improved substantially, and the RMSEA was still acceptable. Hence, reflecting on the chi-square statistic in light of the large sample size, a wide variety of other indices were included to assess model adequacy. The SEM literature states that, as a minimum, RMSEA, CFI, and SRMR should be reported in combination with chi-square [48]. Using multiple fit indices provides a more holistic view of goodness of fit, accounting for sample size, model complexity, and other considerations relevant to the study.

Conversely, the RMSEA estimate has demonstrated lower values with large sample sizes [56, 57]. For an acceptable fit, RMSEA should be ≤ 0.080 [36, 47, 48] or ≤ 0.10 [43], while estimates ≤ 0.050 suggest a good fit. Looking at Model-1, the RMSEA along with SRMR were acceptable and almost good (0.059, 0.052, respectively), while the CFI and TLI were too low. Concerning CFI and TLI, including a cross-loading item (6_D_) along with a correlated error term between the items 2_D_ and 12_D_ improved these fit indices as well as the total model fit. Consequently, low reliability and content validity seemed to cause low values for CFI and TLI.

Theory guided the inclusion of the cross-loading and the correlated error term. It is rational that feeling cheerful (item 6) and simultaneously feeling anxious is a contradiction. To *feel* both cheerful and anxious at the same time is unrealistic. In contrast, people may say, “I still enjoy the things I used to enjoy” (item 2) despite occasionally feeling anxious. The same logic goes for item 4 (“I can laugh and see the funny side of things”) and 12 (“I look forward with enjoyment to things”). To feel cheerful is a feeling, an experience here and now, while being able to ‘enjoy the things that I used to enjoy’ as well as being able to ‘laugh and see the funny side of things’ are not necessarily something a person feels in the moment. These are more general future aspects, such as possibilities or attitudes. Thus, these can go together with having anxiety from time to time. Therefore, we did not allow cross-loadings to the anxiety construct for these items.

### Dimensionality (H_1_)

Concerning the dimensionality of the HADS, the two-factor model undoubtedly showed the best fit to the present data; the dimensionality of the HADS questionnaire stood out to be unquestionable supporting H_1_. The two factors were properly correlated. However, the original two-factor solution did not reveal a good fit. Thus, H_1_ was only partly supported.

### Reliability (H_2_)

The second hypothesis (H_2_) concerned the reliability of the HADS. All items were significant. Largely, the items revealed good loadings (shown in Fig. 1) accompanied by good multiple-squared correlations (R^2^) demonstrating good reliability. Nevertheless, particularly three items belonging to the depression construct (8_D_,10_D_,14_D_) demonstrated low factor loadings and, thus poor reliability, explaining very little of the variance in the construct. These three items caused a low reliability coefficient for depression, while anxiety displayed good reliability. Hence, H_2_ was not fully supported.

### Construct validity (H_3_)

H_3_ tested the construct validity, which concerns whether the set of measured items reflects the theoretical latent construct those items are designed to measure. Hence, it deals with the accuracy of measurement involving psychometric evidence of convergent and discriminant validity [58]. A measure is said to process convergent validity if independent measures of the same construct converge or are highly correlated [49]. Usually, researchers do not have data on two different, e.g., depression scales scored by the same sample: this represents a frequent problem connected with convergent validity. However, measures that theoretically are predicted to correlate significantly with depression might be used. The present study included measures of overall global QOL to test for convergent validity, which was supported by a significant correlation in the expected direction.

Testing discriminant validity, H_4_ stated that HADS correlates significantly and negatively with QOL, while H_5_ expected anxiety and depression to perform as two distinct concepts. Discriminant validity specifically measures whether constructs that theoretically should not be related to each other are, in fact, significantly unrelated. In psychometrics, discriminant validity, also termed divergent validity, indicates that the results obtained by the scale (here HADS) do not correlate too strongly with measurements of a similar but distinct trait; two tests reflecting different constructs should not be strongly related to each other. If they are, we cannot be sure they are not measuring the same construct. Accordingly, discriminant validity indicates the extent of difference between two constructs. The complementary concept to divergent validity is convergent validity; both are forms of construct validity. Hence, a high correlation (higher than 0.40) [59] between HADS and QOL would indicate that the measures substantially overlap and do not behave as clearly distinct constructs [49]. Moreover, a high correlation between anxiety and depression would indicate that the two factors were measuring much of the same trait: this would give a good internal consistency (Cronbach’s alpha and composite reliability) but blur the dimensionality. In this study, the anxiety and depression factors performed like distinct concepts supporting the discriminant validity. Simultaneously, the factor correlation between anxiety and depression was highly significant, supporting convergent validity [49]. The convergent and discriminant validity was further supported by significant correlations in the predicted direction for anxiety and depression towards QOL, supporting hypothesis H_4_.

### Content validity – a vital aspect of construct validity (H_3_)

Content validity is a central aspect of construct validity. Reliability and content validity represent interrelated measurement properties. In fact, despite good reliability, content validity might be poor. Contrariwise, validity cannot be good if reliability is low [49]. Item 8_D_ concerns *“I feel as I’m slowed down”* demonstrated low reliability and, thereby poor validity. In the present sample, with a mean age of 76.5 years, most individuals outside an active work-life have lots of time to adjust to a slower pace of life. Possibly, ‘feeling slowed down’ does not correspond well to older home-living adults’ daily experiences in relation to depression. This item did not perform to be a valid or reliable indicator of depression in this population. Moreover, about 50% of the participants reported physical or mental long-term illness, injury, or loss of function in daily life. Relevantly, a slower pace of life might seem natural and not necessarily an indicator of depression [7].

Likewise, item 10_D_, *“I have lost interest in my appearance,”* did not communicate well with these older adults, indicating low reliability and content validity. Losing interest in one’s appearance did not act as a valid indicator of depression in this population. Losing interest in one’s appearance may be reasoned by the inevitable age-related changes they experience rather than as a symptom of depression. Moreover, item 14_D_, *“I can enjoy a good book or TV program,”* also stood out as an unreliable indicator of depression. Plausibly, being old, enjoying a good book, or watching TV does not relate to depression. Living in your seventies-eighties-nineties, passive leisure activities are everyday activities that are useful as restoration time after active leisure activities and are related to QOL [60]. Reading books might be more demanding due to a decline in sight as well as fatigue. Consequently, item 14_D_ did not explain any variance in the depression construct and thus misbehaved as a valid indicator for the depression construct.

These findings are consistent with previous studies among nursing home residents without cognitive impairment [37] and hospitalized older adults [33], where the same three items were troublesome among older adults in Norwegian care facilities. In older ages, for the first time in their life, retired adults can slow down. Also, due to a decline in age-related reserve capacity and fear of falling, the most common fear in older adults [61], many older adults may be forced to a slower pace. Doing passive activities such as watching TV or reading may also be a consequence of having a chronic medical condition and multimorbidity, which is associated with anxiety and depression [62]. Hence, the wording of the items 8_D,_ 10_D_, and 14_D_ should be carefully considered to improve reliability.

Furthermore, the former validation study among older adults in nursing homes [37] also involved a cross-loading for item 6_D_ to anxiety, as well as highly significant error variances between items 2_D_ and 12_D_. Surprisingly, community-dwelling older adults living at home (the present study), nursing home residents (two different samples giving an approximate *N* = 500; mean age 84.5 and 86 years) [37], and hospitalized older adults (*N* = 484; mean age 80.7 years) [33] respond similarly findings of the HADS-D items.

Summarized, construct validity and reliability of anxiety were good. Conversely, the depression construct revealed low validity and reliability, which are interrelated measurement properties. Exclusively, content validity includes the extent to which elements of a measurement scale are appropriate and characteristic of the specific construct for a certain assessment purpose [49]. In this study, content validity concerns whether the 14 HADS items and the two-factor dimensionality precisely represent anxiety and depression in this population. Besides, evidence of face validity can be considered as one aspect of content validity [49]. High face validity of an instrument increases its use in practical situations via ease of use, proper reading level, clarity, and appropriate response formats. Thus, to improve content validity and thereby also reliability for the depression factor, qualitative studies could be applied to get closer to the actual content of depression, investigating what might be the most essential indicators of depression among community-dwelling older adults. Based on such novel evidence, the three troublesome items could be formulated in a more valid format.

### Strengths and limitations

A notable strength of this research is the empirical examination of the HADS, which has not been tested previously in a community-dwelling older population of 70 + using CFA in Norway. Also, the large sample size is a strength, allowing the possibility to randomly split the sample into two different samples, including 595 community-dwelling older adults each.

Although the older adults were selected randomly in two subsamples, we cannot state that the sample represents the community-dwelling older adults in the actual city since 3181 of 4667 declined participation. In addition, those excluded from this present study were older and had less education. Hence, in the view of representativity, we assume that the present sample may be disrupted, not representing all community-dwelling older adults.

## Conclusion

This study showed that the two-factor structure assessing symptoms of anxiety and depression is unquestionable. In conclusion, when we included a cross-loading item (6_D_) along with a correlated error term between item 2_D_ and 12_D_, a good to acceptable measurement reliability was demonstrated, and construct validity was supported.

However, concerning internal consistency, the original version of HADS revealed a good reliability coefficient for anxiety but a poor estimate for depression; items 8_D_, 10_D_, and 14_D_ stood out as unreliable and invalid indicators for depression in this population. The depression factor includes several items that revealed low reliability (low loadings followed by low R^2^), explaining the minimal variation of the depression construct in this population. Consequently, the depression factor demonstrates low reliability among older community-dwelling people aged 70 + . Therefore, to be valid indicators of depression among community-dwelling older adults, these items need to be rewritten and informed by qualitative studies exploring relevant aspects of depression among older adults living at home.

## Data Availability

The data supporting this study's findings are available from HUNT Databank. Still, restrictions apply to the availability of these data, which were used under license for the current study and are not publicly available. Data are available from the authors upon reasonable request to heidi.sivertsen@ntnu.no and with permission of HUNT Databank: https://www.ntnu.edu/hunt/databank. The Trøndelag Health Study (HUNT) invited persons aged 13—100 to four surveys between 1984 and 2019. Comprehensive data from more than 140,000 persons have participated at least once, and 78,000 persons' biological material is collected. The data are stored in the HUNT databank. HUNT Research Centre has permission from the Norwegian Data Inspectorate to store and handle these data. The key identification in the database is the personal identification number given to all Norwegians at birth or immigration, while de-identified data are sent to researchers upon approval of a research protocol by the Regional Ethical Committee and HUNT Research Centre. To protect participants’ privacy, the HUNT Research Centre aims to limit data storage outside the HUNT databank and cannot deposit data in open repositories. HUNT databank has precise information on all data exported to different projects and can reproduce these on request. There are no restrictions regarding data export approved applications to HUNT Research Centre. For more information: Åsvold BO, Langhammer A, Rehn TA, et al.,. Cohort profile update: The HUNT Study, Norway. Int JEpidemiol. Published 17. May 2022.

## Abbreviations

CFA: Confirmative factor analysis
CFI: Comparative Fit Index
CSDS: Clinically significant depressive symptoms
HUNT: The Trøndelag Health Study
MDD: Major depressive disorder
MI: Modification indices
RMSEA: Root Mean Square Error of Approximation
SEM: Structural equation modeling
SRMR: Standardized Root Mean Square Residual
TLI: Tucker-Lewis Index
